# Effect of the Innovative Running Shoes With the Special Midsole Structure on the Female Runners’ Lower Limb Biomechanics

**DOI:** 10.3389/fbioe.2022.866321

**Published:** 2022-06-06

**Authors:** Fengqin Fu, Lianming Guo, Xunfei Tang, Jiayu Wang, Zhihao Xie, Gusztáv Fekete, Yuhui Cai, Qiuli Hu, Yaodong Gu

**Affiliations:** ^1^ Faculty of Sports Science, Ningbo University, Ningbo, China; ^2^ Doctoral School on Safety and Security Sciences, Óbuda University, Budapest, Hungary; ^3^ Science Laboratory, Innovation center of Xtep Co., Ltd., Xiamen, China; ^4^ Savaria Institute of Technology, Eötvös Loránd University, Budapest, Hungary

**Keywords:** innovation shoes, shoe drop, running, biomechanics, women

## Abstract

The study aimed to research the effects of innovative running shoes (a high heel-to-toe drop and special structure of midsole) on the biomechanics of the lower limbs and perceptual sensitivity in female runners. Fifteen healthy female runners were recruited to run through a 145-m runway with planted force plates at one peculiar speed (3.6 m/s ± 5%) with two kinds of shoe conditions (innovative running shoes *vs*. normal running shoes) while getting biomechanical data. The perception of shoe characteristics was assessed simultaneously through a 15-cm visual analog scale. The statistical parametric mapping technique calculated the time-series parameters. Regarding 0D parameters, the ankle dorsiflexion angle of innovative running shoes at touchdown was higher, and the peak dorsiflexion angle, range of motion, peak dorsiflexion velocity, and plantarflexion moment on the metatarsophalangeal joint of innovative running shoes during running were significantly smaller than those of normal running shoes (all *p* < 0.001). In addition, the braking phase and the time of peak vertical force 1 of innovative running shoes were found to be longer than those of normal running shoes (both *p* < 0.05). Meanwhile, the average vertical loading rate 1, peak vertical loading rate 1, peak braking force, and peak vertical force 1 in the innovative running shoes were lower than those of the normal running shoes during running (both *p* < 0.01). The statistical parametric mapping analysis exhibited a higher ankle dorsiflexion angle (0–4%, *p* < 0.05), a smaller knee internal rotation angle (0–6%, *p* < 0.05) (63–72%, *p* < 0.05), a decreased vertical ground reaction force (11–17%, *p* = 0.009), and braking anteroposterior ground reaction force (22–27%, *p* = 0.043) for innovative running shoes than normal running shoes. Runners were able to perceive the cushioning of innovative running shoes was better than that of normal running shoes. These findings suggested combining the high offset and structure of the midsole would benefit the industrial utilization of shoe producers in light of reducing the risk of running injuries for female runners.

## Introduction

Participants in the half marathon have grown rapidly. The number of participants has increased from 300,000 in 1990 to nearly 2 million in 2013, and over 60% were female runners (Wilder, 2014). It had been recorded that the number of traditionally shod runners who landed with a rearfoot strike (RFS) was more than 80% (Hasegawa et al., 2007; Larson et al., 2011). Commonly running-related injuries such as tibial stress fractures, patellofemoral pain, and plantar fasciitis were linked to the high loading rates and impact transients during rearfoot striking (Milner et al., 2006; Pohl et al., 2009; Davis et al., 2010).

It was related to the fact that male and female runners have significantly different physiological characteristics and sports running posture. A greater active hip internal rotation, vertical ground reaction force (GRF), accessible vertical torque, peak hip flexion angle, and negative work were displayed in females than in males (Ferber et al., 2003). Women had a greater ratio of hip-width to femur length, which resulted in greater hip internal rotation. In addition, women exhibiting higher Q angles increase lateral quadriceps pull on the patella. It would exacerbate patellar tenderness or recurrent lateral patellar subluxation conditions, which induced a higher incidence of patellofemoral joint pain (Horton and Hall, 1989). Tendon stiffness might be associated with the regular use of high-heeled shoes in some females, which led to higher hypertrophy and shortening of the Achilles tendon, a higher pre-activation amplitude of the peroneal muscle, and greater gluteus maximus muscle activation (Baur et al., 2010; Csapo et al., 2010; Napier et al., 2018). Also, 76% of knee pain was found in women. In addition, it was stated that female runners would be twice compared to males as likely to sustain certain running injuries like the aforementioned sports injuries (Taunton et al., 2002). Therefore, it was essential to design running shoes according to female athletes’ biomechanics and body structural characteristics.

Running shoe manufacturers have focused on cushioning, stability, and motion control while making shoes for female runners to diminish running injuries (Malisoux et al., 2016). It was previously proved that reducing the impact forces by wearing a more cushioned shoe may release stress on the musculoskeletal tissue (James et al., 1978; Lieberman et al., 2010). Footwears such as the thickness of the midsole and heel-toe drop (HTD) have been considered in studies of young athletes that could influence a runner’s performance, particularly in cushioning (Hasegawa et al., 2007; Sinclair et al., 2012; Chambon et al., 2015; Nigg et al., 2015). Increasing the midsole thickness could protonate the runner’s effective leg length, such as the Nike Vaporfly 4%, which has a 31-mm heel height (Allen and Kurihara, 1982). It could decrease energy loss for the runner by increasing an effective leg length of 8 mm (Pontzer, 2007; Hoogkamer et al., 2018). In addition, some researchers also figured out that the effect of midsole thickness was about 1% for running economy (Tung et al., 2014).

The HTD would increase with the increased thickness of the heeling material. Recently, the HTD as a critical feature in the shoe design has been linked to running injury risk (Malisoux et al., 2016). Several authors from the biomechanics view had researched the effect of different HTDs. It was reported that a 4-mm HTD induced a higher vertical loading rate than 8 and 12 mm HTD, and the lower limb biomechanics performance of a 4-mm HTD was not similar to barefoot running (Richert et al., 2019). During the investigation, there was no specific adaptation in spatiotemporal variables and kinematics between the three kinds of shoes (0 mm HTD, 6 mm HTD, and 10 mm HTD) (Malisoux et al., 2017). It also found that heel-toe drops (4 and 12 mm) did not directly affect the spatiotemporal parameters of the running cycle in female runners (Gijon-Nogueron et al., 2019). It was still being debated that the thickness of the rearfoot over 45 mm was associated with some gait troubles such as postural disorders and changing spatiotemporal parameters because it would modify the muscle balance up to muscle overuse and strain injuries (Kim et al., 2011; Barkema et al., 2012; Mika et al., 2012; Silva et al., 2013). This was reported that it was essential to affect the center of pressure that the height of the rearfoot had to be more than 2.5 cm (Chien et al., 2013). The vertical loading rate and the associated transient peak reduced as the shoe drops grew. Above all, few shoe manufacturers have made running shoes with an over 12-mm HTD or according to female runners’ characteristics. It was worth researching whether increasing the HTD by more than 12 mm can significantly reduce the impact force or not. Samir et al. (Hessas et al., 2017) asked the volunteer to test barefoot or equipped with three types of stiffness with the same lift height of 20 mm. They found no significant influence of material stiffness on the anterior-posterior displacement of the center of pressure and metatarsal pressures, but it obviously affected the calcaneus’s peak pressure. Furthermore, it was insufficient to investigate the effect of different HTDs on lower limb biomechanics and perceptual sensitivity.

Thus, this study was targeted to investigate how the biomechanics of the lower limb changed when wearing a pair of innovative running shoes (IRS) with a 16-mm HTD which has three layers of the midsole (upper and lower layers were for cushioning, and the middle layer was for support) and to investigate the biomechanics difference between IRS and normal running shoes (NRS). According to the previous literature, it was hypothesized that 1) greater ankle dorsiflexion at touchdown and a lower vertical loading rate would be found in the IRS model than in NRS. 2) The IRS would induce increasing the joints’ moment in the sagittal plane in comparison to the NRS.

## Methods

### Participants

Prior to the study, the sample size of the current study was calculated *via* G*Power 3.1.9.7 (effect size = 0.8, αvalue = 0.05, and power value = 0.8) (Kang, 2021). Also, fifteen female runners [mean (SD) age: 39.00 (10.09) years, height: 1.58 (0.37) m, weight: 50.34 (3.24) kg, and BMI: 20.14 (1.28) kg/m^2^] joined in this research. All participants were recruited from the Xiamen running club and identified themselves as rearfoot strike runners. Participants were uninjured in the lower extremities for at least 6 months before data collection, had a minimum weekly running mileage of 30 km, and could run 10 km in less than 70 min. Participants’ average running experience was 3.53 (0.15) years, their current running exposure was 35.67 (11.48) km/week, and they had an average 10-km time of 72.26 (2.19) minutes. All participants had been confirmed in foot size (EU 37 ± 0.5) by the using Brannock Device (The Brannock Device Co., Syracuse, NY, USA.) before the official test.

### Experimental Footwear

**FIGURE 1:**
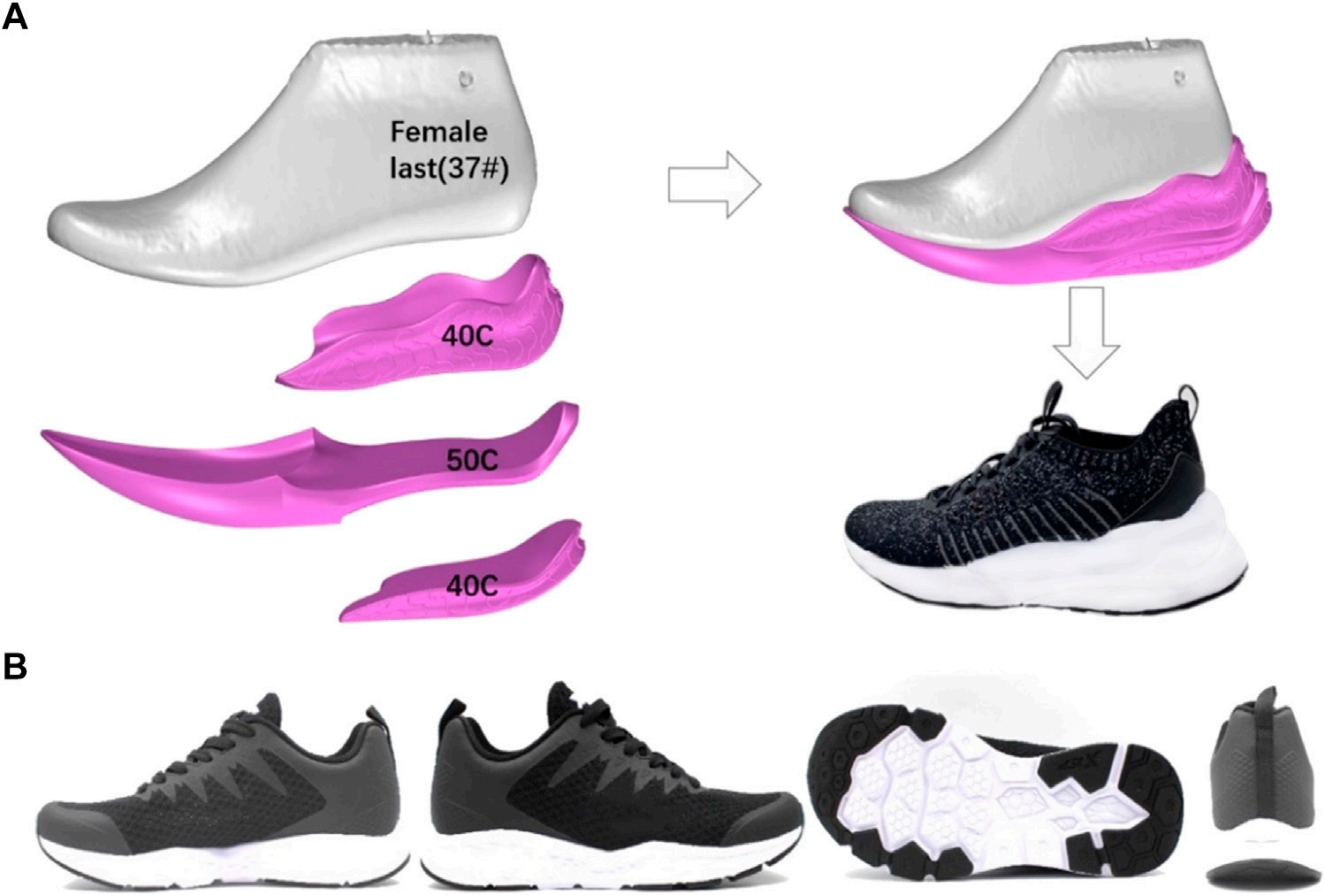
Picture of the IRS prototype used during running **(A)** and NRS **(B)**.

There were two kinds of experimental footwear (innovative running shoes: IRS and normal running shoes: NRS) which differed in their offset, mechanical midsole hardness, rearfoot impact, and forefoot flexion properties used in this research (Figure 1).

The mechanical impact measurement considered the final five impacts from 30 repetitive impacts by using an impact tester (Brentwood, NH, USA) on the experimental shoes with a drop height of 5.0 mm and a drop mass of 8.5 kg (ASTM F19976-13, 2022)**.**

The shoe longitudinal bending stiffness (LBS) and energy return were measured by fixing the forefoot area in the location of 70% foot length (heel to toe) and then bending at 45° by applying a dynamic shoe flexor device (Brentwood, NH, USA) (ASTM F911-85, 1994; Isherwood et al., 2020). All shoe conditions featured an XTEP SoftPad Lite HD foam insole with a forefoot and rearfoot thickness of 5.0 mm. All characteristics of shoes are shown in Table1.

**TABLE 1:** Characteristics of the experimental shoe condition.

Measurement method	Characteristic	Running shoe models	
		IRS	NRS
Basic information	Mass(g)	260.9	205.0
	Rearfoot thickness (mm)	32	18
	Forefoot thickness (mm)	16	11.5
	Offset (mm)	16	6.5
	Rearfoot width (mm)	81.67	80.6
	Forefoot width (mm)	95.6	100
	Midsole material	EVA	EVA
	Midsole hardness (Asker C)	Up to 40 C	55 C
		middle 50 C	
		under 40 C	
	Outsole material	Rubber	Rubber
	Outsole hardness (Asker)	62 A	62 A
Rearfoot impact	Peak acceleration(g)	9.9	13.7
	Energy return (%)	56.61	64.53
Forefoot flexion	Peak torque (Nm)	13.49	9.79
	Stiffness (Nm/deg)	0.307	0.169
	Energy feedback (%)	24.78	27.14

### Experiment Protocol and Procedures

#### Biomechanical Data Collection and Processing Process

The data collection methodology was carried out as in the previous research (Isherwood et al., 2020). Participants performed eight valid right foot rearfoot strike running trials per shoe condition on a 145-m concrete indoor running loop. A valid trial was one within the specified velocity range (3.6 m/s ± 5%) and made up of the whole right foot contacting the force plate area. Previously, data collection participants warmed up for about 5 min and were acquainted with the target speed and shoe conditions by running two laps in each shoe condition. Upon failing to match the required speed in the first two laps, further familiarization laps were performed as necessary.

In GRF and 3D kinematic measurements, participants ran a set of three consecutive laps and flushed into the floor force plates (combined dimensions 270 × 60 cm, 1,000 Hz (AMTI, Watertown, MA, USA)) in each shoe condition. The test sequence of shoes was randomized for each participant. The two-timing gates 8 m far from the middle force plate were used to record the running speed (Smart speed, Burbank, CA, USA) set 8 m apart, centering the middle force plate. Right leg kinematics were collected at 250 Hz and were ordered using a 10-camera motion analysis system in a capture volume of 4.0 × 1.0 × 1.5 m (Vantage 5, Vicon, Metrics Ltd., Oxford, UK). The marker set was according to the calibrated anatomical systems technique (Cappozzo et al., 1995). The right thigh, the right shank, and the right foot (forefoot and rearfoot) were defined as segments by attaching retro-reflective markers of 14 mm in diameter on the skin of the right and left anterior superior iliac spine (ASIS), the right and the left posterior superior iliac spine (PSIS), the right greater trochanter, the medial and lateral epicondyle of the femur, and the medial and lateral malleolus, as well as attached to the shoe, representing the first and fifth metatarsal heads and second toe. Four marker tracking clusters were attached to the lateral side of the thigh and the lateral side of the lower leg (Manal et al., 2000). The extra-reflective markers were added to the distal, proximal heel, and lateral rearfoot, respectively, and were defined as shoe-mounted tracking markers (Heiderscheit et al., 2002). Before data collection, a static trial was conducted; all study procedures about biomechanical data collection were performed in the XTEP science laboratory. In the trial, valid data could be used when the first impact peak and shoe ground angle more than zero appeared. We used the Vicon Nexus 2.7 and Visual3D systems (C-Motion, Germantown, MD, USA) to process the collected experimental data. A fourth-order low pass Butterworth filter was used with a cut-off frequency of 100 Hz (kinetic) and 10 Hz (kinematic) (Isherwood et al., 2020). The XYZ Cardan sequence was used to calculate lower limbs’ kinematic and kinetic data, in which X represents flexion-extension, Y represents abduction–adduction, and Z represents internal-external rotation (Hennig et al., 1993). The angle, the angular velocity, the ground reaction force, and the work of the hip, the knee, the ankle, and the MTP joints of the right lower limb were measured during the stance phase using Visual3D (C-Motion, Germantown, MD, USA). The stride length (SL) was calculated as the anterior-posterior displacement of the right heel marker during two consecutive heel-strike events. The loading rate was calculated as the slope of the vertical GRF between 20 and 80% of the period from heel strike to impact force. All vertical GRF variables were calculated based on the recommendations by Ueda et al. (2016).

#### Subjective Perception

Testing took place simultaneously with biomechanical data collection, with participants filling in on the questionnaire immediately after completing the eight successful trials required for the respective shoe condition. Runners assessed six perception variables (shoe weight, fit, arch support, cushioning, stability, and over preference) on a questionnaire that had been repeatedly highlighted in some articles (Sterzing et al., 2015; Tay et al., 2017; Bishop et al., 2020). Also, a 15-cm visual analog scale (VAS) was carried out; these have been previously applied for running footwear assessment (Mündermann et al., 2002; Mills et al., 2010; Kong et al., 2015; Sterzing et al., 2015). Participants were shown and explained both variables before each shoe condition during the initial familiarization and data collection.

#### Statistical Analysis

For 0D parameters, such as spatiotemporal parameters, average loading rate 1, peak loading rate 1, peak vertical force 1, joint moment, and some kinematics parameters, Shapiro–Wilk tests were adopted for normality distribution. Permutation non-parametric tests were chosen with MATLAB (The MathWorks, Naticks, MA) when the null hypothesis of the normality test was rejected. Paired t-tests were applied when appropriate. Statistics 0D parametric tests were processed by SPSS (24, IBM. Corp, Armonk, NY, USA). Effect sizes (Cohen’s d) were displayed for all statistical tests (0.2 < Cohen’s d < 0.5 = small effect, 0.5 < Cohen’s d < 0.8 = medium effect, and Cohen’s d > 0.8 = large effect).

A statistical parametric mapping (SPM) technique was used to assess the time series parameters such as one-dimensional (1D) kinematic and force trajectories (Pataky et al., 2015; Besson et al., 2019). SPM paired t-tests were performed on shoe effects for every 1D parameter (Nichols and Holmes, 2002). SPM tests were calculated with SPM1D v0.4 for MATLAB (www.spm1d.org, (Pataky et al., 2015)). The statistical significance alpha levels were set to < 0.05 for all statistical tests.

## Results

The running speed was 3.59 ± 0.27 m/s and 3.61 ± 0.31 m/s for IRS and NRS (*p* = 0.673). Shapiro–Wilk tests revealed that 100% of biomechanical variables and 100% of perception variables were normally distributed (both *p* > 0.05).

### Kinematics Variables

Concerning joint angles at touchdown, the ankle of IRS was at a more dorsiflexed position (*p* = 0.023) (Table 2), with no significant changes of the knee, hip flexion, and ankle inversion at the beginning of the contact ground being reported than that of the NRS.

**TABLE 2:** Mean values (±SD) for the main 0D parameters in IRS and NRS.

Variable	IRS	NRS	P	Cohen’s d
Contact time (ms)	205.9 ± 18.1	204.5 ± 17.5	0.079	0.177
Braking phase (ms)	117.3 ± 16.5	108.1 ± 9.4	0.019^a^	0.264
Push-off phase (ms)	90.9 ± 15.5	96.5 ± 11.8	0.061	0.061
Step frequency	187.8 ± 9.2	185.7 ± 9.1	0.410	0.529
Step length(m)	2.18 ± 0.07	2.17 ± 0.06	0.400	0.476
Average loading rate 1 (BW/s)	78.4 ± 20.6	99.9 ± 24.4	0.005^a^	0.606
Peak loading rate 1 (BW/s)	106.7 ± 35.6	169.4 ± 32.2	0.000^a^	0.850
Peak vertical force 1 (BW)	1.93 ± 0.27	2.12 ± 0.27	0.002^a^	0.548
Time to peak vertical force 1 (ms)	39.1 ± 11.0	28.9 ± 5.0	0.001^a^	0.560
Peak braking force (BW)	0.42 ± 0.08	0.46 ± 0.07	0.003^a^	0.362
MTPJ peak plantarflexion moment (Nm/kg)	1.56 ± 0.27	1.80 ± 0.37	0.047^a^	0.298
MTPJ peak dorsiflexion angle (°)	16.2 ± 5.5	20.6 ± 3.8	0.002^a^	0.273
MTPJ ROM in the sagittal plane (°)	18.0 ± 2.7	19.7 ± 3.0	0.000^a^	0.551
MTPJ peak dorsiflexion velocity (°/sec)	352.9 ± 48.2	396.0 ± 55.5	0.000^a^	0.448
MTPJ negative work in the sagittal plane (J/kg)	0.06 ± 0.02	0.07 ± 0.03	0.383	0.071
MTPJ positive work in the sagittal plane (J/kg)	0.004 ± 0.001	0.005 ± 0.002	0.172	0.133
Ankle negative work in the sagittal plane (J/kg)	0.44 ± 0.07	0.46 ± 0.08	0.225	0.267
Ankle positive work in the sagittal plane (J/kg)	0.46 ± 0.07	0.46 ± 0.07	0.267	0.225
Peak ankle plantarflexion moment (Nm/kg)	2.36 ± 0.27	2.21 ± 0.24	0.196	0.187
Peak knee flexion moment (Nm/kg)	2.84 ± 0.31	2.69 ± 0.44	0.168	0.128
Ankle dorsiflexion angle at contact (°)	11.8 ± 5.2	9.4 ± 3.7	0.023^a^	0.546
Peak ankle eversion angle (°)	11.0 ± 4.6	10.2 ± 3.3	0.296	0.097
Peak ankle eversion velocity (°/sec)	299.9 ± 92.9	292.5 ± 51.8	0.557	0.557

a Note: showed a significant effect between IRS and NRS; GRF was normalized to body weight (B.W.).

As for running during stance time, the peak MTPJ dorsiflexion angle and peak MTPJ dorsiflexion velocity of IRS during running were significantly lower than those of NRS (all *p* < 0.001) (Table 2). In the frontal plane, there was no significant difference between IRS and NRS regarding the peak ankle eversion angle and peak ankle eversion velocity. The MTPJ range of motion (ROM) of IRS in the sagittal plane was significantly smaller in comparison with that of NRS (*p* < 0.001) (Table 2); there were no effects of wearing experience on ROM on the ankle, knee, and hip joints in this plane. Ankle ROM (in-eversion) showed no apparent difference between IRS and NRS.

**FIGURE 2:**
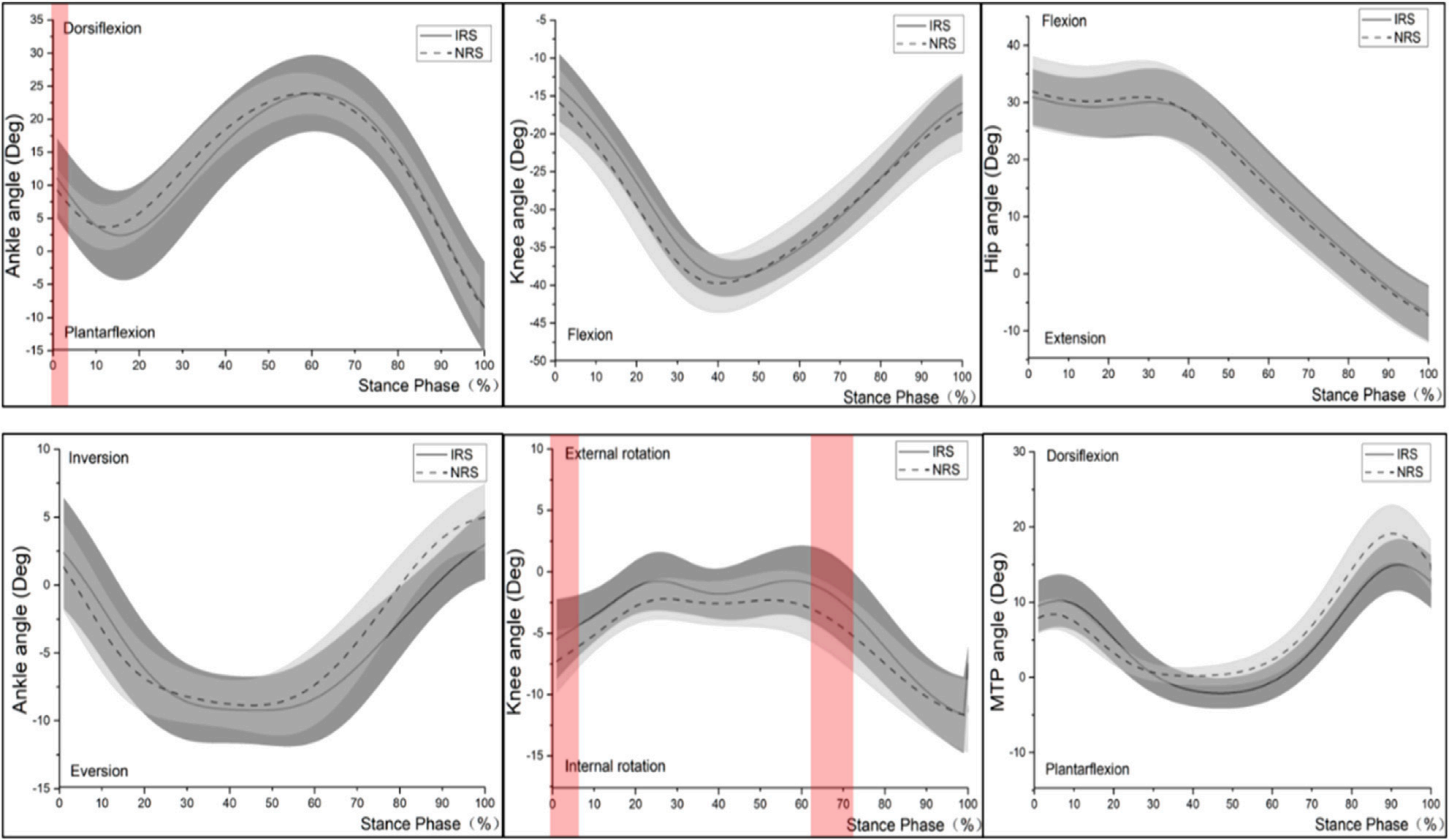
Lower limb joint angles time-normalized. Note: the red horizontal bars within the figure during corresponding periods represent significant shoe effects (SPMT-paired) between IRS and NRS.

The SPM analysis showed a significantly higher ankle dorsiflexion angle for the IRS than for the NRS between 0 and 4% of the stance time (*p* < 0.05). There was a smaller knee internal rotation angle for the IRS from 0 to 6% and from 63 to 72% of stance time than that of NRS (both *p* < 0.05). No significant angle difference between shoe conditions was found around the hip joint (Figure 2).

### Kinetics

There was no significant effect between IRS and NRS on the contact time and push-off phase, yet the braking phase and the time of peak vertical force 1 of IRS were found longer than those of NRS (*p* = 0.019; *p* = 0.001). It induced a certainly lower average vertical loading rate 1 (95%CI (IRS: 67.97–88.83; NRS: 87.55–112.25)) and peak vertical loading rate 1 (95%CI (IRS: 88.68–124.72; NRS: 153.10–185.70)) (both *p* < 0.001). Meanwhile, a lower peak braking force (95%CI (IRS: 0.38–0.46 BW; NRS: 0.42–0.50 BW)) and peak vertical force 1 (95%CI (IRS: 1.79– 2.07 BW; NRS: 1.95–2.26 BW)) were present in the IRS in comparison to the NRS during running (both *p* < 0.001). There was no shoe effect on the step frequency and step length (*p* = 0.410; *p* = 0.400). A lower peak MTPJ plantarflexion moment (95%CI (IRS: 1.42 to 1.70 Nm/kg; NRS: 1.61 to 1.99 Nm/kg)) was found for IRS than NRS (*p* = 0.047). Meanwhile, no significant difference was found in the joints’ work and peak moment (the ankle and knee) in the sagittal plane (Table 2).

**FIGURE 3:**
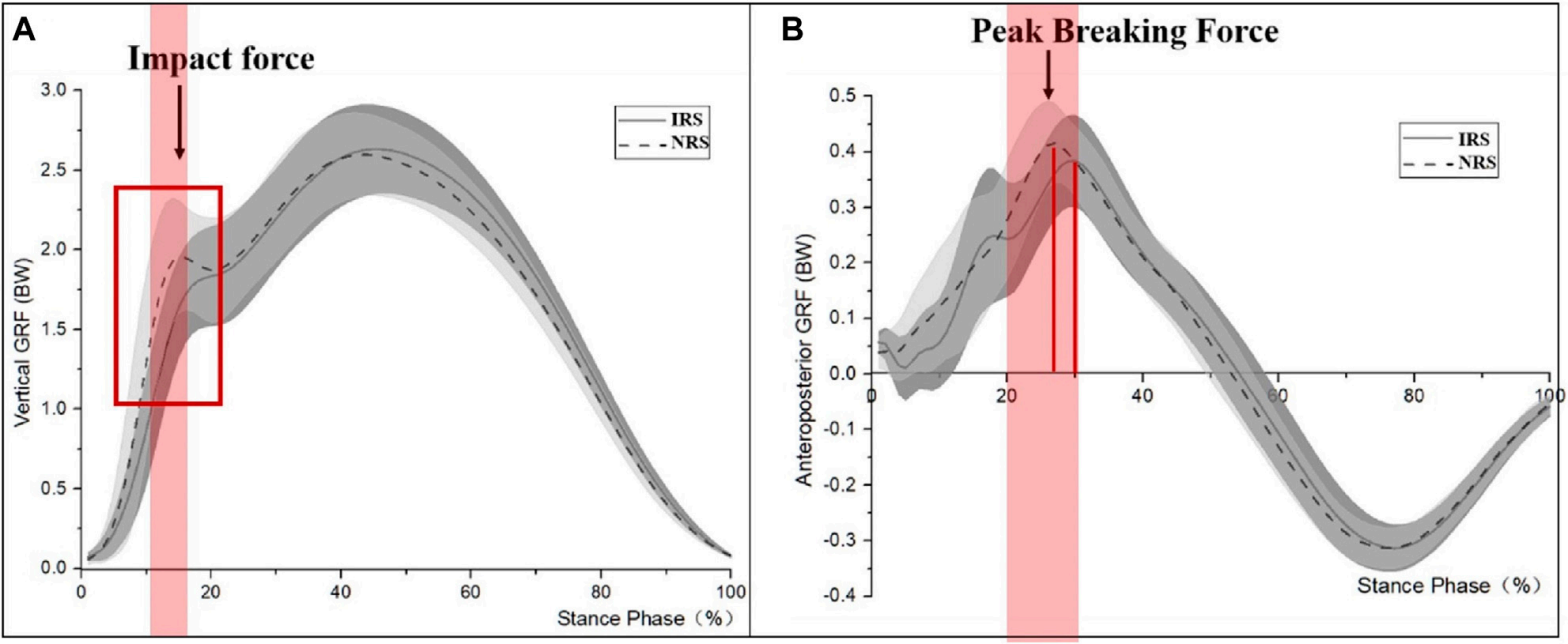
Mean vertical and anteroposterior ground reaction force-time and weight-normalized [**(A)** Vertical GRF, **(B)** anteroposterior GRF]. Positive and negative values are braking and propulsive forces. Standard deviations are presented by white and gray shaded areas. The red horizontal bars within the figure during corresponding periods represent significant shoe effects (SPMT-paired) between IRS and NRS.

**FIGURE 4:**
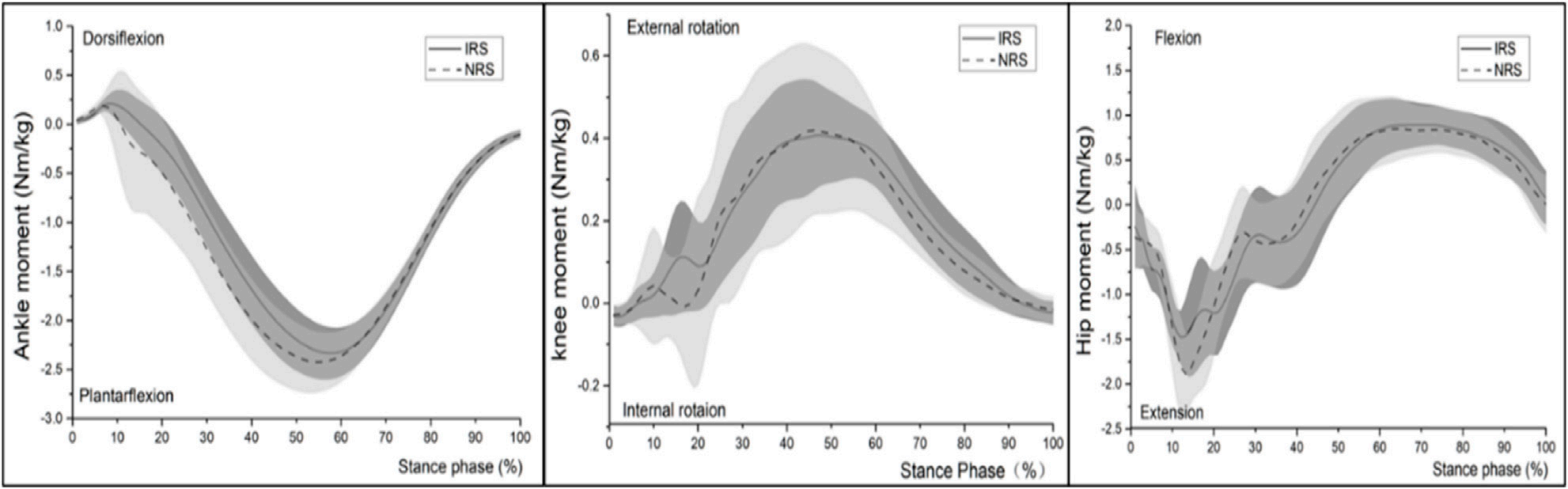
Lower limb joint moment time- and weight-normalized.

The SPM test exhibited a significant effect of shoes on the vertical and anteroposterior components of the ground reaction force (both *p* < 0.001). The IRS decreased vertical GRF from 11 to 17% of the stance phase (*p* = 0.009) (Figure 3A) and decreased the braking anteroposterior GRF from 22 to 27% of the stance phase (*p* = 0.043) compared to the NRS (Figure 3B). No significant moment differences between shoe conditions were reported at the ankle, knee, and hip levels (all *p* > 0.05) (Figure 4).

### Subjective Perception

**FIGURE 5:**
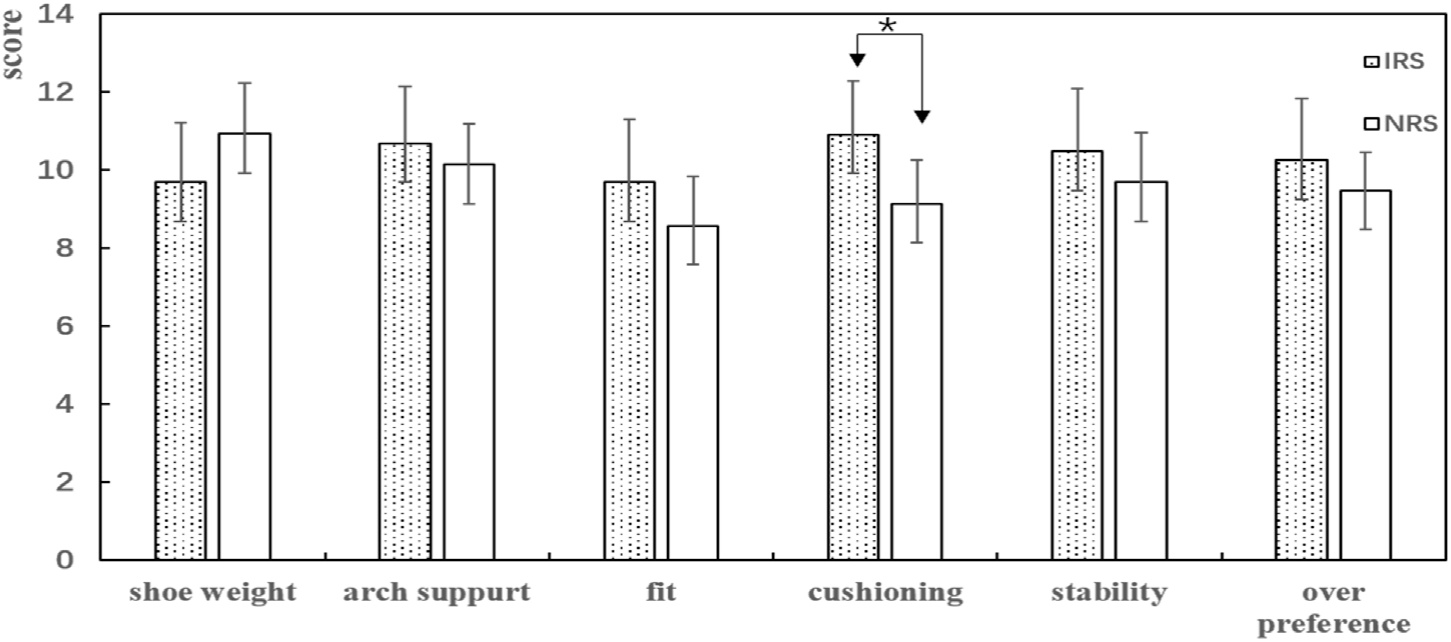
Mean and standard deviations for subjective perception were displayed (higher value and better performance). Note: * showed a significant effect between IRS and NRS.

Runners did not figure out the apparent difference between the two kinds of shoe conditions about the shoe weight, arch support, fit, stability, and over preference. Still, most of them thought that the cushioning of IRS was significantly better than that of NRS (Figure 5).

## Discussion

This study was targeted to clarify the consequence of IRS on female runners and compare the biomechanical difference between these kinds of running shoes during the stance phase. It also aimed to get the longitudinal bending stiffness (LBS) of the forefoot and peak acceleration of the rearfoot by mechanical testing and then on the biomechanical characteristics while running at 3.6 m/s.

According to the first hypothesis, the IRS would improve the shoe cushioning by increasing the HTD and height of the rearfoot during landing. In line with this behavior theory, the mechanical testing indicated a lower peak acceleration in the IRS (9.9 g) than the NRS (13.7 g), which induced significantly biomechanical changes, particularly during the braking phase, like a longer braking phase and the time to peak vertical force 1. Meanwhile, a certainly lower vertical force transient (average and peak) and peak vertical force 1 were present in the IRS in comparison to the NRS during running (all *p* < 0.001). The SPM test exhibited that the IRS decreased vertical GRF from 11 to 17% of the stance phase (*p* = 0.009) (Figure 3A) and decreased braking anteroposterior GRF from 22 to 27% of the stance phase (*p* = 0.043), as compared to the NRS (Figure 3B). All the results were consistent with previous studies (Chambon et al., 2015). It was reported that the vertical loading rate and the associated transient peak increased when the shoe drop decreased. In other words, increasing the shoe drop was the benefit of improving the cushioning of the running shoes.

As for the braking force, a reduced peak braking force was present in the IRS in comparison to the NRS during running. Some authors argued that the peak braking force (higher value) was associated with the risk of injury hazards such as iliotibial band syndrome and should be considered a target for gait retraining interventions (Napier et al., 2018). In other words, IRS in this study can effectively reduce the risk of lower extremity injuries than that of the NRS (Davis et al., 2010; Zadpoor and Nikooyan, 2011; van der Worp et al., 2016; Napier et al., 2018).

During the braking phase, the SPM analysis also showed a significantly higher ankle dorsiflexion angle for IRS, as compared to NRS between 0 and 4% of the stance time, which was consistent with previous reports (Paquette et al., 2013; Chambon et al., 2014; Breine et al., 2017). Horvais and Samozino (2013) confirmed a positive correlation between the shoe drop and shoe ground angle at touchdown in rearfoot runners. For example, the foot/ground angle at touchdown increased when the shoe drop increased.

It should be pointed out that the stability of the shoes is a particularly critical issue when increasing the HTD over 12 mm. In this study, there was no noticeable difference in the running posture at touchdown between the two kinds of shoes, except the ankle of IRS had a more dorsiflexed. In addition, there was no significant difference between IRS and NRS in some stability parameters such as the peak ankle eversion angle, peak ankle eversion velocity, and ankle ROM in the frontal plane in this study. This may be related to the ability of female runners to adapt to high heels and also induced by the special hardness composition of the midsole in IRS. From subjective perception perspectives, runners did not figure out the obvious difference between the two kinds of shoe conditions regarding stability, but most of them thought that the cushioning of IRS was better than that of NRS, which was consistent with our biomechanical results. In addition, there was a smaller knee internal rotation angle for IRS than for NRS from 0 to 6% and from 63 to 72% of stance time (both *p* < 0.05) (Figure 2). It was associated with the structure of the midsole with three layers (upper and lower layers are for cushioning, and the middle layer is for support) which played the role of motion control. Motion-control shoes were beneficial to reduce knee internal rotation in runners with over-pronation, no matter whether fresh or fatigued, which may assist pronated runners in maintaining their stability throughout a fatiguing running (Cheung and Ng, 2007; Lilley et al., 2013; Jafarnezhadgero et al., 2019). Referring to the second hypothesis, as previously shown, a higher net flexion moment on the knee joint or ankle with a higher heel-to-toe offset might increase the strain around the joint (Kerrigan et al., 2009). It was worth noting that a 16-mm shoe drop in this study did not cause significant changes in joints’ (knee and ankle) torque, which would be associated with the midsole’s three layers to modify the stress around the joints (ankle and knee). In addition, the effective limb length of the leg as a strut drives the scaling of locomotor cost for a runner, which was the leg length with the HTD when running with shoes (Allen and Kurihara, 1982). The HTD of the IRS was 9.5 mm more than that of NRS which could protonate the runner’s effective leg length, in other words, increased the force arm of the knee joint, offsetting the influence of the ground reaction force, which might be the reason why no significant difference was found in the knee joint moment (Allen and Kurihara, 1982; Pontzer, 2007; Hoogkamer et al., 2018). This study suggested that it was essential to combine the HTD and hardness of the midsole into account when designing a shoe to improve cushioning and reduce the risk of injuries.

From the perspective of running economy, mechanical testing proved a larger LBS in the IRS (0.307 ± 0.01 Nm/deg) than in the NRS (0.169 ± 0.01 Nm/deg), which induced significant modifications to the running biomechanics during running. Several authors figured out that increasing the stiffness of running shoes might cause a series of biomechanical changes, such as a lower MTPJ range of motion, peak MTPJ dorsiflexion angle, peak MTPJ dorsiflexion velocity, and peak MTPJ plantarflexion moment (Roy and Stefanyshyn, 2006; Willwacher et al., 2013; Smith et al., 2014; Oh and Park, 2017; Barnes and Kilding, 2019; Hunter et al., 2019). Those results were in line with this study. In other words, IRS could improve the running performance by increasing the LBS compared to that of the NRS. Studies had found that increasing LBS improved athletes’ running efficiency ranging between 1 and 2% (Fredericson and Misra, 2007).

### Limitations and Conclusions

Referring to the limitations, the experimental shoes differed in the heel-to-toe offset height and shoe properties. Future investigations should only modify the shoe drop or hardness components of the midsole. In addition, motion-control shoes prevented exacerbated fatigue-related increases in pronated female runners (Dempster et al., 2021; Xiang et al., 2022). It is also extremely important to take the muscle activation of the lower limb and fatigue during running into account, when wearing a running shoe with the midsole’s three layers and 16-mm shoe drop.

Special innovative running shoes had two key points: the heel-to-toe drop of 16 mm and the second was the special hardness component of the midsole. It was the first time to explore the mechanism of the shoe drop reaching 16 mm on running biomechanics. This research added new insights into the mechanism of shoe drops on the female runner. Compared with normal running shoes, innovative running shoes in this study could effectively improve the cushioning and propulsion performance and play the role of motion control. In addition, the female running shoe with a 16 mm shoe drop did not cause significant joint (knee and ankle) torque changes in this study.

## Data Availability

The original contributions presented in the study are included in the article/Supplementary Material; further inquiries can be directed to the corresponding author.
